# Urban park restorative effects in an integrated model of positive emotion and leisure involvement moderation

**DOI:** 10.1038/s41598-025-19451-3

**Published:** 2025-09-30

**Authors:** Dan Yang, Siqi Xie, Ranpeng Su, Weiwei Wu

**Affiliations:** 1 Dan Yang, School of Economics and Management, Chengdu Sport University, Chengdu, 641418 China; 2 Siqi Xie, School of Economics and Management, Chengdu Sport University, Chengdu, 641418 China; 3 Ranpeng Su, School of Economics and Management, Chengdu Sport University, Chengdu, 641418 China; 4 Weiwei Wu, School of Economics and Management, Chengdu Sport University, Chengdu, 641418 China

**Keywords:** Urban parks, Environment type, Restorative effect, Regulating effect, Environmental sciences, Environmental social sciences, Health care

## Abstract

Urban parks are critical spaces for residents to alleviate stress and restore psychological well-being. To investigate the differential impact mechanisms of built and natural environments on residents’ restorative effects and their psychological moderating pathways, this study utilizes the survey data from five urban parks in Chengdu, and applies the questionnaire survey method to analyze the mechanism and contextual conditions of the role of different environmental types on the restorative effects of residents through hierarchical linear regression. It further analyzes the moderating effects of residents’ positive emotion and leisure involvement in the above process; and provides empirical reference value for constructing urban leisure scenes with high restorative effects. The result show that in the influence process of built environment to restorative effect, the moderating effect of leisure involvement is greater than positive emotion. While in the influence process of natural environment to restorative effect, the moderating effect of positive emotion and leisure involvement are similar. Both built environment and natural environment can positively influence the residents’ restorative effect. Residents’ positive emotion and leisure involvement have positive moderating effects on this influence process. The results of this research can be a reference to create an urban recreational environment with restorative effect.

## Introduction

With the rapid progression of urbanization, the pace of residents’ lives and work has accelerated, leading to a gradual increase in associated psychological stress and health risks. This has resulted in research on restorative environments capable of promoting psychological recovery gaining increasing attention^1^. As crucial restorative spaces within cities, urban parks provide essential venues for residents to release negative emotions and restore physical and mental functioning. Although early research tended to suggest that natural environments possess stronger restorative effects than built environments^2^, the urban environment is inherently an integrated system combining both natural and built environments. A growing number of evidence indicates that built environments that align with residents’ preferences and needs can also generate significant restorative effects^3^. Therefore, how to maximize the restorative effects of urban environments in urban planning and leisure management has become an important research topic.

Existing research exploring the psychological mechanisms underlying the restorative effects of urban parks has primarily focused on the mediating role of psychological factors in these effects^4,5^.For example, Li Yihe (2025) found that perceived restorative quality effectively alleviates negative emotions. However, relatively less attention has been paid to how individual differences in psychological states may moderate this process. Individuals in a positive emotional state may exhibit higher sensitivity to the same restorative environment, thereby experiencing recovery more readily or intensely. An individual’s leisure involvement – that is, the depth of participation in and emotional investment in leisure activities – may also significantly influence their proactive seeking and utilization of the environment’s restorative elements, consequently amplifying the restorative effects facilitated by environmental perception.

Thus, the core objective of this study is to integrate positive emotion and leisure involvement as moderating variables into the research framework on the restorative effects of urban parks. By constructing a comprehensive model incorporating the moderating effects of emotion and involvement, this research aims to reveal more deeply the mechanisms through which individual differences operate in the restorative effect process within urban parks. This will provide a more comprehensive theoretical explanation for understanding “why the same environment yields different restorative outcomes for different individuals” and offer an empirical basis for tailored park design and management strategies.

## Literature review and research hypotheses

### Restorative effects of urban park environments

The restorative effects of environments on residents have been empirically validated. In September 2016, a symposium titled *“Exploring Potential Pathways Linking Green Spaces to Health”* was held in Helsin Town, Munich, Germany. Experts from diverse fields and disciplines, including environmental and social epidemiology, environmental psychology, forestry, geography, remote sensing, and urban planning, engaged in interdisciplinary discussions and reached a consensus. They advocated for heightened attention to potential biological, psychological, and social mechanisms underlying these effects^6^. Accordingly, this study adopts the physiological, psychological, and social health benefits proposed in these recommendations as indicators to measure environmental restorative effects.

Common metrics for assessing restorative effects include hospitalization duration, medical electromyography (EMG) values, analgesic dosage, emotional tests, self-reported mood states, and self-rated health assessments^7^. Although medical monitoring instruments offer higher precision in capturing physiological indicators (e.g., brainwaves, cardiac responses), which better reflect physiological restoration, their application in outdoor environments is often impractical due to logistical challenges and time-consuming deployment. While such instruments demonstrate high internal validity in controlled laboratory experiments, their ecological validity diminishes in real-world outdoor settings. Current wearable medical devices, aside from providing relatively accurate heart rate monitoring, exhibit limited reliability for other physiological metrics such as blood pressure and blood glucose, while additional physiological functions remain underdeveloped. Consequently, self-assessment methods for evaluating the restorative effects of leisure environments enable rapid and effective data collection.

Previous studies have explored the restorative effects of environments primarily through the lens of environmental psychology theories, including the *Stress Recovery Theory (SRT)* and the *Attention Restoration Theory (ART)*. The *Stress Recovery Theory* posits that individuals are subjected to various social and environmental stimuli in daily life, eliciting psychological and physiological stress responses of varying intensities. Prolonged exposure to such stimuli can lead to negative emotions such as stress, anxiety, and tension. Natural environments can facilitate physiological recovery and psychological relaxation^8^. And the *Attention Restoration Theory* proposes that natural environments can alleviate cognitive fatigue resulting from the prolonged use of directed attention by engaging involuntary attention. This process serves to restore directed attentional capacity and cognitive functioning^1^, commonly using restorative perception scales to measure urban parks’ restorative effects. The attainment of environmental restorative effects is a sequential and interactive process: individuals begin with physiological relaxation, which subsequently triggers emotional and attentional recovery, along with broader effects Urban park environments provide residents with diverse restorative effects encompassing emotional, physiological, attentional, and cognitive dimensions^9^. These include governance and rehabilitative functions, mental stress alleviation and fatigue reduction, physical health enhancement, emotional refinement cultivation, and social interaction facilitation^10^. In park settings, residents are more likely to experience positive emotions, vitality, and life satisfaction^11^. Urban parks enhance residents’ physical activity levels, alleviate mental stress, reduce fatigue^12^, and foster social interactions, thereby strengthening community cohesion and social inclusivity^13^.

However, existing research on the restorative effects of urban parks has predominantly focused on natural elements (e.g., lawns, shrubs, trees, flowers, and water bodies), with findings indicating variations in restorative effects across these elements^14^. In contrast, limited attention has been paid to the restorative potential of built environments. Recent studies have begun to examine the role of recreational and sports facilities in enhancing restoration^15^ and the optimization of restorative effects through spatial design^16^. Empirical evidence suggests that higher naturalness in urban parks does not necessarily yield superior restorative effects, as natural elements alone are insufficient to fully explain restorative effects^17^. Therefore, integrating natural and built environments may maximize the restorative benefits of urban parks.

### Pathways to enhance restorative effects

Perceived restorativeness is a critical pathway to improving restorative effects. Current measurements of perceived restorativeness in China predominantly rely on the *Perceived Restorative Scale (PRS)*, which includes four dimensions: *being away*, *fascination*, *compatibility*, and *extent*^18^. However, due to cultural, economic, and institutional differences between China and Western contexts, existing adaptations of the PRS exhibit limitations in explanatory power^19^. This scale primarily reflects Western cultural expressions and conceptual understandings. Western studies focus on how physical features of natural environments (e.g., greenness ratio, water area, openness) influence restoration^20^. Research indicates Western restorative environment assessments emphasize functional characteristics and aesthetic qualities. Chinese studies prioritize social functions of built environments, developing localized measurements based on Harting’s PRS by incorporating more social relational factors^21^.Furthermore, restorative perception measurements have been mainly applied in tourism destination studies. Post-pandemic, with decreased resident travel radius, urban parks have become crucial daily leisure and healing spaces. Therefore, this study’s exploration of restorative environment perception effects on restoration—considering both built and natural environments in urban parks—is essential.

Natural environments contain diverse factors that contribute to restorative effects, with vegetation and water bodies being among the most significant. Therefore, forest parks are commonly used as important sites for relevant research both domestically and internationally due to their abundant vegetation^22,23^. Early studies on environmental restoration employed experimental methods such as exposing participants to color slides, revealing that landscapes featuring water and vegetation captured attention and interest more effectively than urban scenes^24^. Similar research demonstrated that waterside environments in cities and urban woodlands offer stronger restorative experiences^25^. Different types of environments—such as urban streets, urban parks, managed woodlands, and wild forests—exhibit varying restorative effects^26^. These findings suggest that the restorative effects of urban public spaces depend on individual perceptions, needs, and physical characteristics of the environment. Beyond visual experiences, auditory elements of restoration have also been investigated. Experimental studies confirm that natural sounds, such as bird songs and flowing water, enhance restorative perceptions and significantly promote individual recovery and positive experiences^27^; different sound types^28^and sound combinations^29^produce distinct restorative effects. Additionally, high-quality natural environments often provide clean air, benefiting residents’ health. Existing research confirms that people experience stronger environmental restorative effects on days defined as having good air quality^30^.

Additionally, leisure activities contribute to restorative effects. Studies demonstrate a positive association between leisure activity types and environmental restoration^31^,with duration of engagement also playing a role^32^. Static leisure behaviors (e.g., sitting, meditating) and dynamic activities (e.g., exercising) mediated by urban facilities (e.g., benches, sports equipment) positively influence restorative effects^33^.

Individual differences also influence the enhancement of restorative effects. Emotional preferences and self-regulation positively correlate with restoration^34^, particularly through the mediating role of *place attachment*^35^. Environmental preferences, such as affinity for urban built environments, further shape restorative effects^36^.

### Research hypotheses

Research confirms that environmental impacts on restorative effects exhibit threshold effects and are moderated by contextual factors^37^. Given that positive emotions facilitate restoration^21^and focused attention during environmental interactions amplifies restorative effects^38^, this study hypothesizes that high levels of positive affect and deep leisure involvement enhance the magnitude and ease of environmental impacts on restoration. Specifically, both *positive affect* and *leisure involvement* are posited as positive moderators in the relationship between environments and restorative effects.

Furthermore, based on the literature, both natural elements (e.g., vegetation, water bodies) and built environments (e.g., plazas, pathways) can elicit restorative effects when aligned with residents’ preferences. Thus, this study hypothesizes that both natural and built environments in urban parks positively influence residents’ restorative effects.

**Fig. 1:**
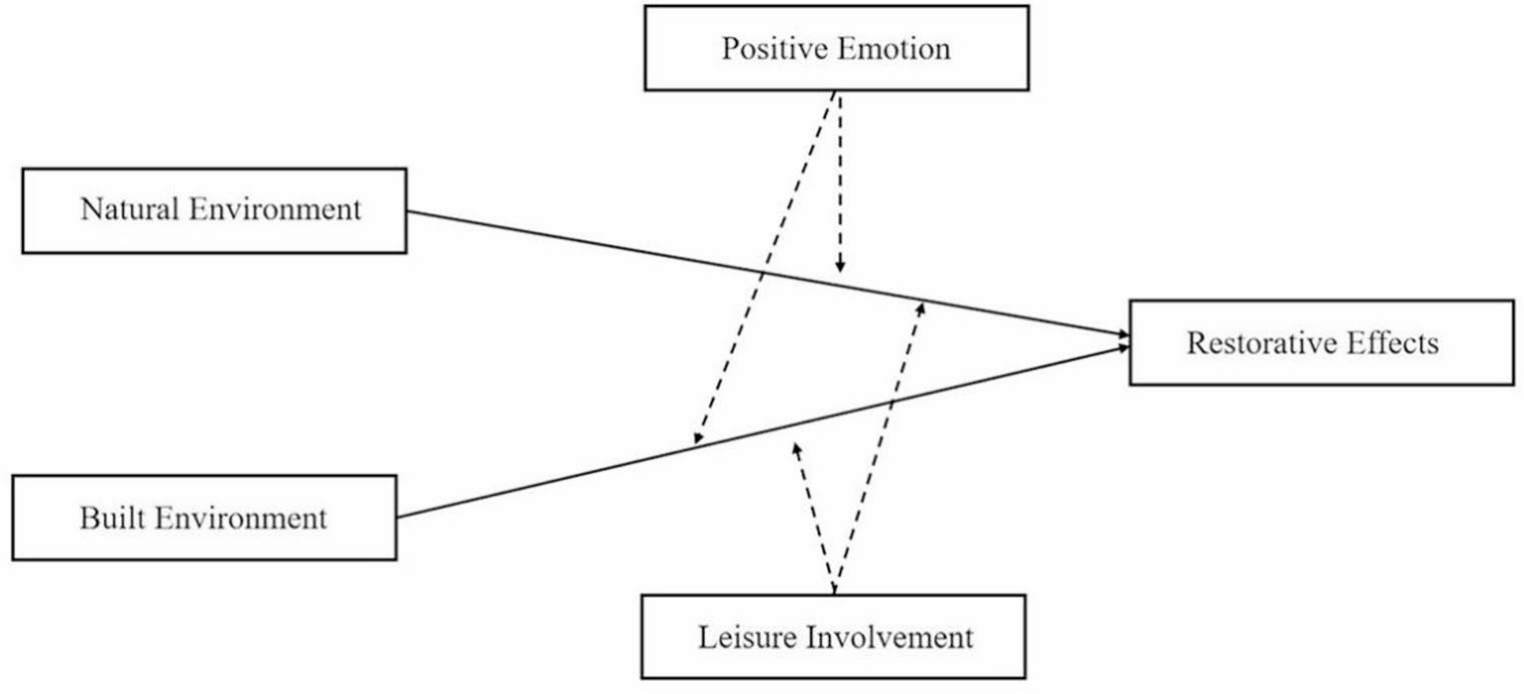
Research hypothesis model.

In summary, this research constructs a conceptual model (Fig. 1) to examine the moderating roles of *positive affect* and *leisure involvement* in the relationship between environmental factors and restorative effects.

Figure 1 Theoretical mechanism explanation: As antecedent variables, both natural and built environments directly promote restorative effects through sensory stimuli and behavioral support—a relationship extensively documented domestically and internationally^10,39^. Furthermore, existing research demonstrates that natural environments influence restorative effects through positive emotion^4^, with both environmental types capable of eliciting positive emotion to generate restorative effects^40^. Regarding leisure involvement, studies adopting tourist perspectives confirm its positive moderating effect on restorative effects^41^; however, research examining this mechanism remains comparatively scarce.

## Research methodology

### Selection of research site

**Fig. 2:**
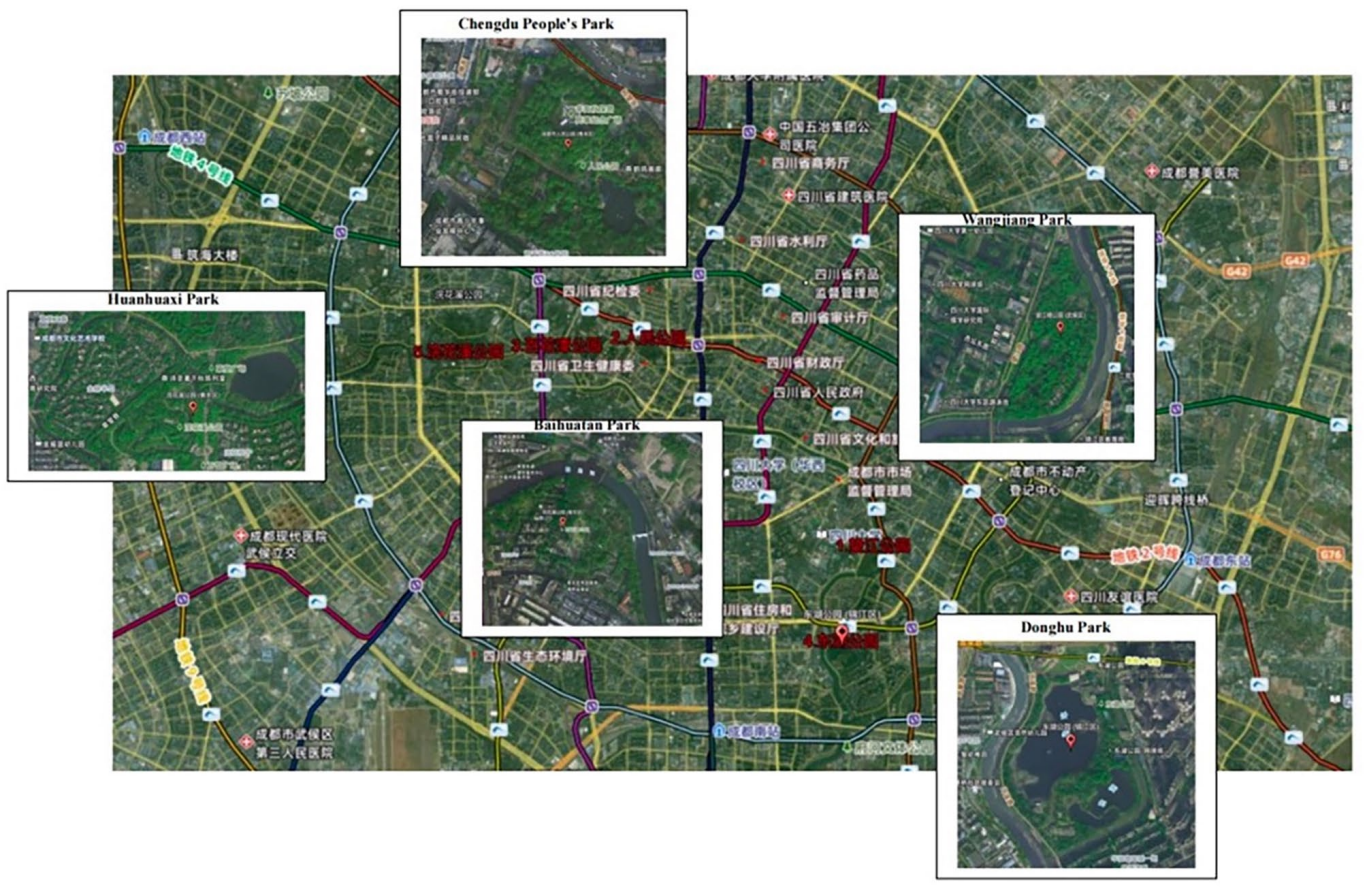
The location of five parks (From OvitalMap V10.3.0, https://zj.eguvtvu.cn/aowei9.html? bd_vid=6768640522005481658).

In 2018, General Secretary Xi Jinping first mentioned the goal of building a “park city” in Chengdu, and in 2020, he again supported Chengdu to build a “park city demonstration area that practices the new development concept”. In recent years, Chengdu has been committed to creating an urban environment in which “going out is a park, and everywhere is a scene”, and has achieved milestones in the construction of a park city. In this study, five parks in Chengdu, namely Wangjiang Park, People’s Park, Baihuatan Park, Donghu Park, and Huanhuaxi Park, were selected for the study based on the principles of uniform distribution of areas, diversified landscape types, long history of construction, and large flow of people (Fig. 2.). The case sites cover different environmental types in Chengdu urban area, which are typical and representative. (Table 1)

**Table 1:** Relevant information of five parks.

The name of the park	Area(hectare)	Landscape features
Wangjiang park	12.53	Natural environment: Centered on bamboo culture, the park boasts China’s richest bamboo resources, forming lush bamboo groves with high vegetation coverage. Adjacent to the Jinjiang River, its water bodies and greenery collaboratively create a humid, refreshing atmosphere. Natural birdsong, rustling bamboo leaves, and localized flowing water blend into an immersive ecological soundscape. Built Environment: Daily scenes feature citizens engaging in morning exercises, tea-drinking, and photography, demonstrating both urban vitality and serving as a cultural showcase.
Chengdu people’s park	11.26	Natural environment: The park features pleasant vegetation (high greenery coverage with renowned ancient trees and over 70 tree species) and beautiful water features (Jinshui Stream, artificial lake, and Goldfish Island forming a waterside system). Its refreshing air and microclimate stem from dense vegetation collaborating with water bodies to cool, humidify, and mitigate urban heat—earning the “urban green lung” title. Natural sounds of insects, birdsong, babbling streams, and rustling lotus leaves interweave into an ecological soundscape. Built environment: Recreational facilities blend history and modernity: century-old Crane Cry Teahouse and Zhenliu Teahouse, lake boats, and East Rockery Waterfall. Activity spaces include the 3,000 m² Xinhai Railway Protection Memorial Square for assemblies and exhibitions. Post-“removing walls to integrate greenery”, enhanced accessibility enables daily scenes like tea-drinking, morning exercises, and exhibition-viewing.
Baihuatan park	9	Natural environment: Baihuatan Park centers on an artificial lake and waterfall system, featuring a millennium-old ginkgo tree and a Sichuan-style penjing garden housing nearly 1,000 diverse bonsai specimens. Ecological soundscapes integrate waterfall murmurs, birdsong, and rustling foliage, while dense vegetation purifies air to establish a humid, refreshing microclimate. Built environment: The park’s core facility—the Sichuan-style Penjing Garden—complements the Bajin Culture Garden (Huì Garden) with nighttime illuminations. Through the Green Integration Project, a 730-meter Xunxiang Trail (fragrance-seeking pedestrian corridor) connects Ginkgo Square to bonsai exhibition zones. The leisure atmosphere highlights citizen activities including bonsai appreciation, teahouse gatherings, and horticultural practices, achieving fusion between cultural heritage and natural immersion.
Donghu park	193.21	Natural environment: Its 185-mu waterbody and native vegetation jointly create a humid, refreshing microclimate. The urban lake ecosystem—with 77.4% aquatic plant coverage—attracts abundant birdlife for habitat and breeding. Built environment: The leisure facilities include a lakeside greenway, meeting residents’ exercise needs, and a bookhouse. The recreational atmosphere combines cultural experiences with physical exercise.
Huanhuaxi park	30	Natural environment: Featuring century-old osmanthus, camphor, ginkgo, hibiscus bamboo, and Wanshu Mountain bamboo groves, the park’s 210,000 m² high vegetation coverage ensures fresh air and microclimate regulation. Its core water bodies—Canglang Lake and Egret Islet Wetland—purify water through ecological embankments, attracting egret habitats. Natural soundscapes blend egret calls, babbling streams, and bamboo rustling. Built environment: The park features a Poetry Avenue with famous verses, a Poetry Allusion Garden with sculptures, the “Rippling Flowers Pavilion” at Canglang Lake, a Sichuan-West Culture Performance Plaza, and the Poet Sage Plaza—providing gathering and activity spaces where poetic elements blend with urban vitality.

### Data collection

This study used the questionnaire survey method to collect research data from August 2020 to November 2020 to measure the restorative effects of the five parks in different environments with the residents who carry out recreational activities in the five parks as the research subjects. Residents engaging in leisure activities across various time periods in five parks were surveyed. The survey was administered by applicable guidelines and regulations and was reviewed and approved by the School of Economics and Management at Chengdu Sport University. Prior to questionnaire distribution, informed consent was obtained from all participants or their legal guardians. Respondents were assured of strict data confidentiality, emphasizing that the collected information would be used exclusively for scientific research purposes without commercial utilization. The participants provide their written informed consent to participate in this study. A total of 300 questionnaires were distributed and a total of 265 questionnaires were returned, with 235 valid questionnaires. Among them, 31.49% were Male; 68.51% were female. As for the distribution of age groups, residents aged 41–55 years old accounted for the largest proportion, 40.62%. In terms of education level, residents with college education or above accounted for the largest proportion, at 55.32 per cent. In terms of monthly income distribution, RMB 4001–6000 accounted for the largest proportion, at 14.47%. In terms of the frequency of outdoor leisure, the number of weekly leisure times of residents is not high, in which the average weekly leisure times are less than once accounted for 32.34%, and the average weekly leisure once accounted for 46.81%. In terms of the average duration length of a single leisure session, the vast Majority of residents spent less than 2 h on a single leisure session; 42.55% of them spent less than 1 h, 45.53% spent 1–2 h, 8.09% spent 2–3 h, and 3.83% spent more than 3 h. (Table 2)

**Table 2:** Summary of demographic characteristics.

Category		Frequency(*N* = 235)	Rercentage(%)
Park	Chengdu People’s Park	43	18.3
	Baihuatan Park	40	17.0
	Wangjiang Park	41	17.4
	Donghu Park	56	23.8
	Huanhuaxi Park	55	23.4
Gender	Male	74	31.5
	Female	161	68.5
Age	Under 18 years	19	8.1
	18–28 years	18	7.7
	29–40 years	28	11.9
	41–55 years	95	40.4
	Over 55 years	75	31.9
Education level	Below junior high	19	8.1
	Junior high school	45	19.1
	High school	35	14.9
	College/Undergraduate	31	55.7
	Postgraduate	5	2.1
Monthly income (RMB)	< 2000	25	53.2
	2001–4000	48	20.4
	4001–6000	32	13.6
	6001–8000	14	6.0
	> 8000	16	6.8
Weekly leisure frequency	< 1 time	76	32.3
	= 1 time	111	47.2
	2–3 times	31	13.2
	> 3 times	17	7.2
Leisure duration per sessiin	< 1 h	100	42.6
	1–2 h	107	45.5
	2–3 h	19	8.1
	> 3 h	9	3.8

### Measurement tools

On the basis of summarizing the results of relevant literature studies at home and abroad, the Mature scales in the existing research Literature were borrowed to measure the Main variables involved in this study. The scale question item indicators were scored on a Likert 7-point scale, with scores ranging from low to high indicating the subjects’ degree of agreement (compliance), where 1 indicates complete disagreement (or compliance) and 7 indicates complete agreement (or compliance).

#### Dependent variable: environmental restorative effects

The measurement scales employed in this study draw from established research by Diette^42^, Maas^43^, Peschardt^44^, Liu Qunyue^45^, and Peng Huiyun^46^among others. These scales broadly encompass three dimensions of environmental restorative effects: physical restoration, psychological restoration, and social restoration. Representative items for physical restoration include *“alleviation of bodily pain/discomfort”*; for psychological restoration, *“reduction in anxiety/depression/stress”*; and for social restoration, *“increased social interactions/reduced loneliness.”*

#### Explanatory variables: natural environments and built environments

Building on these variables, the scales primarily draw upon and adapt instruments developed by Roger (1981)^24^, Korpela (2010)^25^, Van (2014)^26^, and Zhang Yuan (2016)^47^, among domestic and international scholars. This study measures natural environments through four core dimensions: green spaces, water bodies, soundscapes, and air quality.

In addition, in leisure and recreational contexts, individuals interact not only with natural elements like vegetation and water but also with built environmental factors (e.g., facilities, spatial layouts) and social environmental factors (e.g., recreational atmosphere, activities, and social interactions)^48^.Empirical studies confirm that built environments similarly generate restorative effects^49,50^. For built environment measurement, considering simplicity and core relevance, this study operationalizes built environments through three dimensions: recreational sites, facilities, and ambiance.

#### Moderating variables: positive emotions and leisure involvement

Restorative effects are generally positively correlated with individuals’ emotional states, necessitating detailed exploration of the interplay between emotional well-being and restoration across different environmental types^51^. According to theoretical reasoning, heightened positive emotions are more conducive to physical and mental health. This study treats positive emotions as a moderating variable influencing the relationship between environmental factors and restorative effects. The measurement scale draws from Scherer^52^ and Huang Li^53^, including six items such as: “I feel happy during leisure time”,“Leisure activities make me feel fulfilled”, “Leisure activities inspire enthusiasm in me”, “I feel relaxed during leisure time”.

The moderating variable leisure involvement assesses residents’ behavioral engagement in leisure activities rather than inherent traits. This study adapts Chiu et al.’s leisure tourism involvement scale^54^, modifying items to align with urban recreational contexts. Examples include: “The leisure activities here are enjoyable”, “I share my leisure experiences with others”. Three items are used to measure this construct.

#### Control variables

In addition to the influence of natural environmental factors, scholars have also focused on the interference effects of individual characteristics, such as age, gender, household income, leisure time duration, and physical activity levels, on restorative environmental experiences. Studies suggest that demographic variables like gender and age are critical covariates in environmental restoration research, as individual differences significantly impact psychological and physiological restorative effects in natural settings^17,55,56^.

Building on prior research, this study incorporates both personal characteristics (e.g., gender, age, occupation, education level, income) and leisure behavior traits (e.g., leisure frequency, duration) as potential explanatory variables in the analysis of restorative effects. These factors are included as covariates to account for their potential confounding effects.

## Empirical results and analysis

### Common method bias test

To address potential common method bias (CMB) in self-reported surveys, Harman’s single-factor test was conducted. Exploratory Factor Analysis (EFA) on 235 valid samples revealed that the variance explained by the first unrotated principal component was 23.968%, well below the 40% threshold, indicating no significant CMB in this study.

### Reliability and validity tests

Reliability was assessed using composite reliability (CR) and factor loadings. Validity was evaluated via confirmatory factor analysis (CFA), with convergent validity measured by average variance extracted (AVE) and discriminant validity confirmed by comparing the square roots of AVE values with inter-construct correlations (Tables 3 and 4).

**Table 3:** Scale items, reliability, and validity metrics.

Latent variable	Measurement items	Factor loadings	CR	AVE	Cronbach’s α
Positive emotion	Proud	0.811	0.888	0.575	0.883
	Happy	0.832			
	Fulfilled	0.838			
	Enthusiastic	0.520			
	Relaxed	0.764			
	Energetic	0.737			
Leisure involvement	Engaged	0.702	0.879	0.710	0.872
	Enjoyable	0.927			
	Shared experiences	0.882			
Built environment	Atmosphere	0.683	0.827	0.617	0.817
	Venue	0.796			
	Facilities	0.866			
Natural environment	Air quality	0.722	0.842	0.572	0.836
	Vegetation	0.818			
	Soundscape	0.727			
	Waterfeatures	0.755			
Restorative effect	Pain relief	0.637	0.828	0.448	0.824
	Anxiety reduction	0.739			
	Energy boost	0.705			
	Activity improvement	0.675			
	Social interaction	0.689			
	Sleep quality	0.554			

**Table 4:** Discriminant validity: comparison of ave square roots.

Latent variable	AVE	Positive emotion	Leisure involvement	Built environment	Natural environment	Restorative effect
1.Positive emotion	0.575	0.758				
2.Leisure involvement	0.710	0.014	0.843			
3.Built environment	0.617	0.066	−0.044	0.785		
4.Natural environment	0.572	0.009	0.093	0.445	0.756	
5.Restorative effect	0.448	−0.030	0.015	0.516	0.562	0.669

Note: Diagonal values (bold) are square roots of AVE; off-diagonal values are inter-construct correlations.

### Descriptive statistics

#### Heterogeneity analysis of five urban parks

This study selected five representative parks in Chengdu distinguished by diverse locations, high popularity, and frequent resident usage. Aggregated statistical analysis of respondents’ mean perception scores for natural environments across these parks revealed (Table 5): Huanhuaxi Park scored highest (M = 5.5636), followed by Donghu Park (M = 5.2679), while the People’s Park (M = 3.7733) and Baihuatan Park (M = 4.6500) registered the lowest scores. These results indicate stronger natural environment perception in Huanhuaxi and Donghu Parks, contrasting with weaker perception in the People’s Park and Baihuatan Parks. The elevated scores for Huanhuaxi and Donghu Parks primarily derive from their extensive green coverage, abundant aquatic landscapes, diverse botanical configurations, and well-preserved ecosystems. Although the People’s Park and Baihuatan Park possess aesthetically pleasing natural environments, their downtown locations necessitate hosting intensive recreational activities (e.g., square dancing, matchmaking events), which diminish respondents’ perception of natural elements. Huanhuaxi Park and Donghu Park scored significantly higher in the evaluation of natural environmental attributes. This advantage is primarily attributable to their substantially larger land area compared to the People’s Park and Baihuatan Park. The expansive green spaces, abundant water features, and diverse vegetation inherent to larger parks inherently foster a stronger perception of natural environments. Conversely, the lower scores for the People’s Park and Baihuatan Park should not be interpreted as reflecting inferior natural beauty. Rather, their central urban location, historically established robust social functions, and high visitor density have consistently positioned them as central hubs for public activities, social interaction, and leisure. Consequently, their pronounced social character largely overshadows their natural attributes. Visitors primarily experience the urban vitality and local cultural atmosphere characteristic of these locations, which consequently attenuates their perception of the natural environment.

**Table 5:** Respondents’ perception and evaluation of the natural environment in urban parks.

		*N*	Mean	Std. Deviation	Std. Error	95% Confidence Interval for Mean		Minimum	Maximum	Between- Component Variance
						Lower Bound	Upper Bound			
the People’s park		43	3.7733	0.96488	0.14714	3.4763	4.0702	1.25	5.25	
Baihuatan park		40	4.6500	0.78610	0.12429	4.3986	4.9014	2.75	6.50	
Wangjiang park		41	5.2378	0.87848	0.13720	4.9605	5.5151	2.75	7.00	
Donghu park		56	5.2679	1.03995	0.13897	4.9894	5.5464	3.00	7.00	
Huanhuaxi park		55	5.5636	0.96731	0.13043	5.3021	5.8251	3.75	7.00	
Total		235	4.9532	1.12657	0.07349	4.8084	5.0980	1.25	7.00	
Model	Fixed effects			0.94197	0.06145	4.8321	5.0743			
	Random effects				0.31859	4.0687	5.8377			0.47796

Although the natural environment perception scores show notable variations across the five urban parks, a one-way ANOVA was conducted to determine whether these differences attain statistical significance. The analysis revealed statistically significant differences in natural attributes among the five parks (F = 26.176, *P* < 0.001). Subsequent post-hoc multiple comparisons using LSD method identified pairwise differences as presented in Table 6. The results demonstrate that all pairwise comparisons exhibit statistically significant differences (*P* < 0.05), except for the following non-significant contrasts: Huanhuaxi Park vs. Wangjiang Park, Huanhuaxi Park vs. Donghu Park, and Donghu Park vs. Wangjiang Park (*P* > 0.05). This confirms substantial heterogeneity in natural attributes among the five parks, ensuring representative sampling and enhancing the generalizability of research findings. Based on the aforementioned statistical analysis, Huanhuaxi Park, Donghu Park, and Wangjiang Park demonstrate comparable scores in perceived natural attributes, forming a distinct cluster characterized by “prominent natural features.” Conversely, the People’s Park and Baihuatan Park exhibit significantly lower scores than this cluster. For this grouping, visitors’ experiences are predominantly shaped by social activities within the parks. These spaces are perceived as an “urban living room” or “multifunctional plaza”, where their social character overshadows natural attributes. The divergence likely stems from differing design and management paradigms: the first three parks prioritize maintaining and highlighting their “ecological value”, while the latter two emphasize fulfilling citizens diverse and intensive “social activity needs”. The former approach yields higher perceived natural environment scores, whereas the latter—despite serving vital urban functions—objectively diminishes individual perception of “naturalness”.

**Table 6:** Correlation coefficients of main research variables.

Multiple comparisons							
Dependent variable: mean value of natural environment							
	(I) Park	(J) Park	Mean difference (I-J)	Std. Error	Sig.	95% Confidence Interval	
						Lower bound	Upper bound
LSD	Huanhuaxi Park	The People’s Park	1.79038^*^	0.19175	0.000	1.4126	2.1682
		Baihuatan Park	0.91364^*^	0.19574	0.000	0.5280	1.2993
		Wangjiang Park	0.32583	0.19436	0.095	− 0.0571	0.7088
		Donghu Park	0.29578	0.17882	0.099	− 0.0566	0.6481
	Donghu Park	The People’s Park	1.49460^*^	0.19100	0.000	1.1183	1.8709
		Baihuatan Park	0.61786^*^	0.19501	0.002	0.2336	1.0021
		Wangjiang Park	0.03005	0.19361	0.877	− 0.3514	0.4115
	Wangjiang Park	The People’s Park	1.46455^*^	0.20561	0.000	1.0594	1.8697
		Baihuatan Park	0.58780^*^	0.20934	0.005	0.1753	1.0003
	Baihuatan Park	The People’s Park	0.87674^*^	0.20692	0.000	0.4690	1.2845

*. The mean difference is significant at the 0.05 level.

Means, standard deviations, and correlations are summarized in Table 7.. Significant positive correlations were observed between built and natural environments (*r* = 0.399, *p* < 0.01), built environment and restorative effects (*r* = 0.473, *p* < 0.01), and natural environment and restorative effects (*r* = 0.456, *p* < 0.01). Correlation coefficients indicate that restorative effects (dependent variable) exhibit significantly positive correlations with both independent variables—built environments and natural environments. This establishes the prerequisite foundation for subsequent regression model analysis. Furthermore, neither moderating variable (positive emotion, leisure involvement) shows significant correlation with the dependent variable (restorative effects), nor with either independent variable (built environments, natural environments). This pattern confirms both moderating variables are well-suited for the hypothesized model as they demonstrate the essential independence required for testing moderation effects.

**Table 7:** Means, standard deviations, and correlations.

Latent variable	M	SD	Positive emotion	Leisure involvement	Builtenvironment	Natural environment	Restorative effect
1.Positive emotion	2.8468	0.9700	—				
2.Leisure involvement	3.0596	1.1650	0.006	—			
3.Built environment	4.4950	1.2075	0.081	−0.067	—		
4.Natural environment	4.9926	1.1027	0.024	0.081	0.399**	—	
5.Restorative effect	4.5206	1.0210	0.003	0.019	0.473**	0.456**	—

Note: *p < 0.05**p < 0.01,two-tailed tests.

### Hypothesis testing

To examine the direct effects of environmental factors on restorative effects, hierarchical Linear regression was employed. In Model 1 (Table 8), restorative effect was regressed on demographic variables (gender, age, education level, monthly income) and leisure behavior traits (leisure frequency, leisure duration).

Regression results indicated non-significant coefficients for gender, age, education level, and monthly income, suggesting demographic characteristics are insufficient to explain restorative effects. Conversely, leisure frequency (β = 0.197, *p* < 0.01) and leisure duration (β = 0.158, *p* < 0.05) partially explained restorative effects, with the regression model yielding R²=0.103. The regression equation for Model 1 is expressed as:

Restorative Effect = 4.012–0.2479 × Gender + 0.069 × Age − 0.095 × Education Level − 0.037 × Monthly Income + 0.230 × Leisure Frequency + 0.210 × Leisure Duration.

In Model 2, which added built recreational environment and natural recreational environment as independent variables to Model 1, regression results demonstrated significant positive effects of both built environment (β = 0.309, *p* < 0.001) and natural environment (β = 0.315, *p* < 0.001) on restorative effects. The model achieved R²=0.365. These findings confirm the hypothesis that environmental factors positively influence restorative effects. The regression equation for Model 2 is:

Restorative Effect = 1.588 − 0.138 × Gender + 0.024 × Age − 0.083 × Education Level − 0.015 × Monthly Income + 0.205 × Leisure Frequency + 0.112 × Leisure Duration + 0.266 × Built Environment + 0.286 × Natural Environment.

**Table 8:** Regression results.

Variable	Model 1			Model 2			
	B (β)	se	t	B (β)		se	t
Constant	4.012	0.364	11.018^***^	1.588		0.397	3.998^***^
Gender	−0.2479 (−0.112)	0.140	−1.758	−0.138 (−0.063)		0.119	−1.155
Age	0.069(0.051)	0.092	0.753	0.024(0.018)		0.078	0.307
Education	−0.095 (−0.097)	0.065	−1.473	−0.083 (−0.085)		0.056	−1.493
Income	−0.037 (−0.044)	0.056	−0.661	−0.015 (−0.018)		0.048	− 0.313
Leisure frequency	0.230(0.197)	0.083	2.767^**^	0.205(0.176)		0.070	2.917^**^
Leisure duration	0.210(0.158)	0.088	2.386^*^	0.112(0.085)		0.075	1.501
Built environment				0.266(0.309)		0.050	5.334^***^
Natural environment				0.286(0.315)		0.055	5.227^***^
R^2^		0.103				0.365	
F		4.386^***^				16.260^***^	
△R^2^						0.262	
△F						46.618^***^	

Note: *p < 0.05**p < 0.01***p < 0.001.

In the moderation analysis, this study first centered the research variables and utilized the PROCESS 3.5 plugin in SPSS to examine the dual moderating effects of positive emotion and leisure involvement at different levels on the relationship between environmental factors (built vs. natural environments) and restorative effects. The analysis employed Model 2 with a 95% confidence interval and 5,000 bootstrap samples. Restorative effects were set as the dependent variable, while positive emotion and leisure involvement served as dual moderators. Leisure frequency and duration were included as control variables.

For built environments, results indicated significant positive moderation by leisure involvement (β = 0.1131, SE = 0.0524, t = 2.1575, *p* < 0.01) and positive emotion (β = 0.0934, SE = 0.0424, t = 2.2015, *p* < 0.05). The standardized coefficient comparison revealed that leisure involvement exerted a stronger moderating effect than positive emotion. For natural environments, both positive emotion (β = 0.1201, SE = 0.0554, t = 2.1702, *p* < 0.05) and leisure involvement (β = 0.1257, SE = 0.0437, t = 2.8773, *p* < 0.01) showed significant positive moderation, with their effects being comparable in magnitude.

For built environments, simple slope tests revealed that under low positive emotion, leisure involvement significantly moderated the impact of built environments on restorative effects: low involvement (β = 0.1944, *p* < 0.05), medium involvement (β = 0.3033, *p* < 0.001), and high involvement (β = 0.4121, *p* < 0.001). Similar patterns emerged at medium and high positive emotion levels, with progressively stronger effects as moderators increased.

For natural environments, under low positive emotion, leisure involvement showed non-significant moderation at low levels (β = 0.1342, *p* > 0.05) but significant effects at medium (β = 0.2806, *p* < 0.001) and high involvement (β = 0.4271, *p* < 0.001). At higher positive emotion levels, all involvement levels exhibited significant moderation, with the strongest effect observed at high involvement (β = 0.6601, *p* < 0.001).

The Attention Restoration Theory (ART) posits that prolonged use of directed attention leads to mental fatigue, and that exposure to natural environments can assist individuals in restoring attentional capacity and cognitive functioning. Within the pathway through which the built environment influences restorative effects in this study, the individual focuses more on “what” they are doing rather than “where” they are doing it. Leisure involvement emphasizes the degree of an individual’s active participation in leisure activities; consequently, it exerts a stronger moderating effect than positive emotion in this context. The Stress Recovery Theory (SRT) posits that when individuals experience stressful situations, exposure to natural environments facilitates the restoration of emotional and physiological states, returning the mind and body to a healthier equilibrium. Within the pathway through which the natural environment influences restorative effects in this study, the individual focuses more on “where” they are rather than “what” they are doing. Compared to leisure involvement, positive emotion more effectively facilitates restorative effects arising from natural environmental elements such as greenery, water bodies, soundscapes, and air quality within natural environments. Therefore, its moderating effect is more pronounced.

To summarize, the dual moderating roles of positive emotion and leisure involvement were confirmed. Both variables enhanced the positive effects of built and natural environments on restorative effects, with the magnitude of influence escalating as moderators increased. This underscores the synergistic interplay between psychological states and behavioral engagement in shaping environmental restorative benefits.

## Research conclusions and discussion

### Research conclusions

This study, situated within the context of urban park environments, developed a dual-moderator model incorporating residents’ positive emotion and leisure involvement. It systematically investigated the moderating mechanisms of these two psychological characteristics in the formation of environmental restorative effects, innovatively revealing the differentiated roles and synergistic effects of individual psychological factors across different environmental types. The conclusions are as follows:

Firstly, constructing a “dual-path” moderating model of restorative effects, this study confirmed distinct moderating pathways of different psychological variables within built versus natural environments. Pathway 1 is the “Active Construction Pathway” in Built Environments. The study found that within built environments, the moderating effect of leisure involvement is substantially stronger than that of positive emotion. This reveals that the restorative value of built environments stems primarily from their affordance – that is, the possibilities they offer residents for action and participation. However, this potential must be activated and realized through individuals’ active engagement and investment (high leisure involvement). Whether using fitness facilities or participating in cultural exhibitions, the restorative experience does not arise from passive environmental perception but is actively constructed by the individual. This finding significantly expands the traditional research focus on physical environmental attributes, highlighting the decisive role of individual agency in realizing the value of built environments. Pathway 2 is the “Synergistic Co-creation Pathway” in Natural Environments. The study found that within natural environments, the moderating effects of positive emotion and leisure involvement are equally important, demonstrating synergistic enhancement. This deepens the classic Attention Restoration Theory (ART) and Stress Recovery Theory (SRT). Residents’ pre-existing positive emotion acts as a “catalyst”, amplifying the therapeutic effects of natural environments in unconsciously eliciting positive affect and alleviating stress (resonating with SRT). Simultaneously, leisure involvement reflects residents’ high interest and perceived fit with specific activities, enabling them to more fully utilize the natural environment’s fascination and compatibility to restore directed attention (resonating with ART). Therefore, in natural environments, the restorative effect results from the synergistic co-creation of “passive emotional perception” and “active experiential investment”.

Secondly, urban parks possess the unique restorative function of synergistic enhancement between built and natural environments. This study found that, in the context of urban parks, the positive influence of built and natural environments on residents’ restorative effects is comparable. This conclusion supplements the prevalent view in prior research favoring the superior restorative potential of natural environments. This is not to negate the importance of nature but rather reveals the unique character of urban parks as “hybrid ecosystems”. Unlike wilderness parks, the essence of urban parks lies in the high integration of a natural base with built facilities. Within this study’s context, built environments (such as greenways, benches, and fitness areas) do not diminish natural restorative capacity; instead, by providing activity platforms and enhancing accessibility, they significantly augment the efficiency of natural resource utilization and the depth of experience. The two form a relationship of “synergistic effect”. This finding provides a crucial theoretical basis for urban park planning: the core objective should not be simply maximizing natural space, but rather promoting deep human-nature interaction through “human-centered” facility design, thereby maximizing overall restorative benefits.

Thirdly, individual psychological experience holds a “central position” within the urban park context. This study found that demographic characteristics (e.g., gender, age) and external behavioral patterns (e.g., leisure frequency, duration) exhibited no significant effects on restorative outcomes. This series of “non-significant” results underscores the decisive role of intrinsic psychological state (positive emotion) and engagement depth (leisure involvement) in the environmental restorative process. It emphasizes that psychological mechanisms are key to understanding and enhancing environmental restorativeness, driving a shift in environment-behavior research from superficial variables towards deep psychological processes. The findings indicate that the emergence of environmental restorative effects hinges not on “who comes” or “how often they come”, but on the individual’s emotional state (positive emotion) and level of investment (leisure involvement) during participation. This propels a paradigm shift from focusing on external conditions to exploring internal mechanisms, further solidifying the core mediating and moderating status of psychological processes like emotion and motivation within environment-behavior relationships, and providing robust empirical support for the psychological foundations of restorative environment theory.

In summary, by constructing and validating a dual-moderator model, this study systematically reveals that the restorative effects of urban parks constitute a dynamic process actively moderated and constructed by individual psychological characteristics. The research not only deepens the understanding of person-environment interactions but also, through the proposal of the “dual-path model” and the “theory of synergistic restorative environments”, provides novel theoretical perspectives and scientific foundations for future environmental psychology research and urban park planning practice.

### Implications for management

Firstly, recognizing the significant role of built environments in promoting residents’ restorative effects, it is essential to optimize park accessibility and spatial layouts according to leisure needs and preferences, thereby creating healthy recreational scenarios. This includes enhancing provisions of park plazas, greenways, and leisure facilities. Simultaneously, specialized facilities for vulnerable groups (e.g., elderly, disabled individuals) should be expanded. Guided by the “engagement-oriented” principle, the functionality and layout of built environments require optimization. Research confirms that leisure involvement is the primary moderating variable activating the restorative potential of built environments. Consequently, planning should extend beyond basic facilities to enhance their “Affordance” and “Engagement Capacity”. Specific strategies include: (I) Implementing tiered designs for fitness facilities and greenway systems to accommodate diverse activity needs, thereby deepening activity centrality and focus. (II) Employing “immersive spatial design” techniques—through refined acoustic and visual landscapes—to strengthen zoning effectiveness and create distinctive “micro-destinations” that minimize distractions and facilitate flow states. (III) Expanding “universal and barrier-free design” from physical accessibility to perceptual and cognitive inclusivity, ensuring all groups—particularly those with special needs—can participate in park activities with minimal barriers and high engagement.

Secondly, promote “synergistic integration of natural and built environments” in urban leisure planning and design. Research confirms that both natural and built environments contribute equally to restorative effects in urban parks, existing not as independent elements but as functionally complementary and mutually empowering systems. Practically, deep integration should be achieved through bidirectional pathways: “leveraging natural landscapes to enhance built environment experiences” and “empowering natural experiences through artificial facilities”. In natural zones, focus on ambiance creation and emotional resonance; in built facility areas, prioritize functional optimization to stimulate deep engagement and skill development. Within the “natural landscapes enhancing built experiences” strategy, integrate diverse natural elements—such as wetlands, arboreal corridors, and multi-seasonal floral displays—around pathways, water edges, and shaded trails. This creates dynamic-static recreational spaces that amplify visitors’ immersive experiences and psychological restoration. Furthermore, for the “artificial facilities empowering nature experiences” approach, implement tiered pathway systems tailored to distinct natural settings, complemented by regularly guided activities and sporting events. This provides diversified recreational experiences for visitors across involvement levels, elevates perceptibility of natural landscapes, and fosters synergy between natural attraction and functional restoration.

Finally, park operational management must shift from “physical space maintenance” to “psychological experience empowerment”. Given that positive emotion and leisure involvement is intrinsic drivers of restorative effects, park managers should transition from “site administrators” to “experience facilitators”: On the one hand, for positive emotion enhancement, develop diverse activities (sports, music, arts), host greenway cultural festivals, extend visitation duration, and create immersive flow experiences—making park environments accessible, perceptible, and appreciable. On the other hand, for leisure involvement, establish a tiered activity system (introductory, community-interest, and competitive events), leveraging professional guidance to elevate participation skills and experience quality. Critically, differentiated management strategies should be implemented. In natural zones, cultivate serene atmospheres to evoke emotional resonance. In built facilities, optimize functionality and organize activities to stimulate deep engagement and skill development. Through such integrated hardware-software and subjective-objective management innovations, resident agency can be fully mobilized to maximize the conversion of environmental potential into tangible psychological well-being.

### Limitations and future directions

On the one hand, with regard to the questionnaire scale used in this study, although it met the requirements of scale research standards in terms of validity and reliability, there are still possibilities of incomplete evaluation dimensions and biased selection of evaluation indicators. In the future, we can consider absorbing the corresponding latest scale results to further refine, enrich and improve the research design guidance. On the other hand, in the reality of urban leisure, the paths and relationships of various environmental factors on the restorative effects of residents are extremely complex, and there are numerous types of influencing variables in the composition. Due to the limitation of space, this study only focuses on the moderating effect of two factors, namely, positive emotions and leisure involvement, on the restorative effect of environmental influences, and fails to consider other psychological variables that may bring about changes in their influence, as well as intermediate moderating variables in the process of the restorative effect of environmental factors. In the future, a more complex research model combining mediating and moderating roles can be constructed to explore the interactions and explanatory mechanisms among various environmental, individual and leisure organization management factors, so as to expand the depth and breadth of the research on restorative effects in urban parks.

Furthermore, the urban park environmental perception data utilized in this study—while possessing unique historical value as pandemic-onset records from 2020—now exhibit temporal limitations due to the five-year gap. This data currency constraint may affect the contemporary applicability of findings, particularly given accelerated urban park development and smart infrastructure advancements. Consequently, interpretations of restorative effects must cautiously account for evolving environmental variables. Future longitudinal tracking is recommended to verify evolving patterns of restorative effects.

## Data Availability

The datasets generated or analyzed during the current study are not publicly available due to the confidentiality agreements adhered to in our research team but are available from the corresponding author on reasonable request.

## Declarations

All authors have read, understood, and have complied as applicable with the statement on “Ethical responsibilities of Authors” as found in the Instructions for Authors.
